# Engaging citizens to improve service provision for oral health

**DOI:** 10.2471/BLT.22.287944

**Published:** 2022-05-01

**Authors:** Stefan Listl, John N Lavis, Lois K Cohen, Manu Raj Mathur

**Affiliations:** a Department of Dentistry, Radboud University Medical Center, Philips van Leydenlaan 25, 6525 EX Nijmegen, Netherlands.; b McMaster Health Forum, McMaster University, Hamilton, Canada.; c Paul G. Rogers Society for Global Health Research, Bethesda, United States of America.; d Queen Mary University of London, London, England.

Oral health is rarely a priority of health policy agendas,^1^^–^^3^ although the recent World Health Organization (WHO) Resolution on Oral Health (WHA74.5) highlighted that oral diseases affect over 3.5 billion people worldwide and are among the most expensive diseases to treat.^3^ Such diseases particularly affect poorer and marginalized groups and are a key driver of catastrophic health expenditures.^2^ For those without access to essential oral health care, oral diseases can result in reduced performance of essential daily functions, pain and discomfort, and systemic infections; they can also necessitate emergency hospital admission.^1^^,^^2^

Scientists and WHO have repeatedly emphasized the need for oral health systems improvement.^1^^–^^3^ However, progress in oral health systems transformation has been slow. Concrete know-how for spurring evidence- and values-driven action has been lacking for multiple reasons.

First, lack of clarity persists as to what constitutes – from citizens’ perspectives – essential oral care and how it can be optimally governed, funded and delivered. Without comprehensive accountability for citizen values, divergent stakeholder interests complicate rather than address problems. Second, the oral health community remains disconnected from the broader health community. Although oral diseases and noncommunicable diseases share common risk factors and sequelae, oral health systems rarely benefit from innovations in other areas of health care.^1^ Third, a high level of provider influence exists within the oral health policy ecosystem. The traditional business models of organized dentistry often give precedence to private financing approaches, contributing to a culture of reluctance against public governance and delivery arrangements. Fourth, the level of idiosyncrasy is high within the dental research ecosystem. Traditionally anchored biomedical research targets clinical disease management rather than empowering citizens to maintain good oral health.^1^ Dental public health research has been successfully describing problems, but scaling up implementation research to improve oral health is needed.^1^

Therefore, a complex systems problem exists, characterized by limited accountability for incorporating citizen values and research evidence on the effectiveness of oral health programmes and interventions at systems level; for seeking greater integration with broader health systems; and for identifying the critical junctures where path dependencies can be overcome. Inertia and inaction will leave us with a persistent, albeit largely preventable, burden of oral diseases. A different approach is needed to improve oral health systems. 

Evidence-informed deliberative processes provide one promising approach. Citizen panels can gather citizens with diverse backgrounds and experiences to collectively deliberate about key oral health-system problems, options to address them and key implementation considerations. The gathered citizen values can then be used in policy-making processes to counter narrow stakeholder interests that perpetuate a separation of oral health from the broader health system and that block efforts to improve governance, financial and delivery arrangements.

Recent studies describe the role of citizen deliberations for context-specific health policy-making.^4^ Initiatives such as the Evidence-informed Policy Network have successfully demonstrated how deliberative processes can be harnessed to strengthen health systems and get the right programmes and services to those who need them.^5^ Health technology assessment agencies consider evidence-informed deliberative processes relevant.^6^ A recent case study from Ireland shows how citizens can be involved in the development of health system performance assessment frameworks.^7^ Thailand’s co-production concept provides an example of harnessing citizen values for public health policy.^8^

Deliberative processes can also be leveraged at the community level.^9^^,^^10^ These processes are relevant for poorer and marginalized groups that face problems in accessing affordable essential health care. For example, the Institute of Medicine Community Health Improvement Process describes an iterative approach to identify and prioritize, together with citizens, pressing health issues in community settings, and subsequently develop, implement and monitor concrete improvement strategies.^9^

On the clinical practice level, citizens with experience as patients can be involved in deliberations about quality improvement. Iterative reflective learning based on patient feedback can spur quality improvement.^10^

In the oral health context, however, take-up of deliberative approaches as outlined above is currently limited. To ensure access to essential oral health care for everyone without causing financial hardship, the oral health community needs to commit to harnessing citizen values via evidence-informed deliberative processes. Describing the problem without identifying actionable solutions and implementation strategies is no longer an option. Recent developments such as the WHA74.5 Resolution,^3^ the development of a global strategy on tackling oral diseases,^11^ the addition of dental preparations to WHO’s Model List of Essential Medicines, the *Lancet* Commission on Oral Health and the World Dental Federation’s Vision 2030 report^12^ provide unique windows of opportunity.
